# Current Concepts in Fluid Therapy in Horses

**DOI:** 10.3389/fvets.2021.648774

**Published:** 2021-03-29

**Authors:** Naomi E. Crabtree, Kira L. Epstein

**Affiliations:** Department of Large Animal Medicine, University of Georgia College of Veterinary Medicine, Athens, GA, United States

**Keywords:** fluid therapy, fluid administration, crystalloids, colloids, horse

## Abstract

Despite the frequent inclusion of fluid therapy in the treatment of many conditions in horses, there are limited studies available to provide evidenced-based, species-specific recommendations. Thus, equine fluid therapy is based on the application of physiology and extrapolation from evidence in other veterinary species and human medicine. The physiologic principles that underly the use of fluids in medicine are, at first glance, straightforward and simple to understand. However, in the past 20 years, multiple studies in human medicine have shown that creating recommendations based on theory in combination with experimental and/or small clinical studies does not consistently result in best practice. As a result, there are ongoing controversies in human medicine over fluid types, volumes, and routes of administration. For example, the use of 0.9% NaCl as the replacement fluid of choice is being questioned, and the theoretical benefits of colloids have not translated to clinical cases and negative effects are greater than predicted. In this review, the current body of equine research in fluid therapy will be reviewed, connections to the controversies in human medicine and other veterinary species will be explored and, where appropriate, recommendations for fluid therapy in the adult horse will be made based on the available evidence. This review is focused on the decisions surrounding developing a fluid plan involving crystalloids, synthetic colloids, and plasma.

## Introduction

Fluid therapy is a key component in treatment and supportive care of horses with a variety of conditions, especially those with critical illness. Despite its frequent utilization, it remains challenging to make evidence-based treatment recommendations. This is due to the limited number and design of studies related to fluid therapy that have been performed in horses (1). The majority of those that have been published are either experimental (healthy or healthy with induced pathology) or retrospective and, therefore, provide only low-level evidence. While there are a handful of prospective, randomized studies in horses, they are marred by small sample size and often have significant case heterogenicity limiting their strength as well. As a result of the minimal species-specific information available, recommendations for fluid therapy often rely heavily on the lowest levels of evidence - expert opinion, theory, physiology, and extrapolation from other animal species and human medicine.

At the simplest level, fluids are drugs and, as such, have the potential for both life-saving benefits and serious, possibly life-threatening, adverse effects. In order to better align their utilization with their characterization as drugs, human medicine has begun a paradigm shift toward considering their use within the “four Ds” of drug therapy: drug, dosing, duration, de-escalation (2). Treating fluids as any other prescription drug ensures appropriate consideration of options and approaches to maximize their benefits and limit complications. Several current controversies within human medicine can easily be framed within the concept of the “four Ds” and “prescribing” of fluids, involving the choice of route, fluid type, and dose.

Given that equine practitioners must rely heavily on extrapolation, it is not surprising that both recommendations and controversies regarding fluid therapy in horses parallel those in human medicine and other veterinary species. For the purposes of the current review, we will focus on three areas of debate related to the first two D's of fluids: drug (route and type) and dose. **Route of Administration** will discuss when and how to utilize the various routes of fluid administration available in the horse. As with all drugs, the administration route must be determined early on when developing a fluid therapy plan. In horses, the most commonly utilized routes are intravenous (IV) and intragastric (IG). Rectal (PR) fluid administration may be an alternative and has been a topic of recent interest. Once the route of fluid administration is decided, fluid type and dose must be selected. Information on fluid type and dose for IG and PR fluid administration are provided in **Intragastric Fluids** and **Rectal Fluids**, respectively. Following this, **Intravenous Fluids - Crystalloids** and **Intravenous Fluids - Colloids** will discuss the choice of type and dose of IV fluid therapy, with **Intravenous Fluids - Crystalloids** focused on the crystalloids and **Intravenous Fluids - Colloids** focused on colloids. Throughout this review, we aim to delve into the controversies surrounding choosing the most appropriate route, fluid type, and dose and report the most current literature, in order to help the equine practitioner navigate this territory. Where appropriate, recommendations for treatment of the adult horse will be made, based on the available evidence. It should be noted that the present review does not touch on transfusion medicine, nor parenteral nutrition, information for which has been well-reviewed elsewhere (3–9).

## Route of Administration

Factors that must be taken into account when choosing route of administration include those related to the patient's medical conditions, such as the indications and contraindications for fluid therapy associated with the underlying disease and co-morbidities, as well as non-medical considerations, such as patient temperament, owner finances, and the environment. Together, these determine the likelihood that the treatment will be successful and the potential for adverse effects. Table 1 provides a starting point for clinicians when choosing a route of administration, while Table 2 provides common dosing recommendations. Additional details are provided in the relevant sub-sections.

**Table 1:** Factors influencing the choice of route of fluid administration to horses.

	**Indication/reason for fluid administration**				**Medical contraindications**	**Safety**	**Cost**	**Patient and environmental factors**
	**Resuscitation**	**Dehydration**	**Daily requirements**	**Specific problem**				
IV	Route of choice	Appropriate choice—may result in rebound dehydration due to continued natriuresis	Appropriate choice	Hypovolemia; significant or rapid ongoing losses; other (e.g., blood product administration, parenteral nutrition, etc.,)	Anuric and oliguric renal failure, caution with heart failure	Catheter associated complications—thrombosis, thrombophlebitis, air embolism	$$$$	Availability for stalling and close monitoring
IG	Unlikely to be of benefit	Appropriate choice—may be preferred due to less urinary loss when discontinued	Appropriate choice	Additional benefits likely for GI hydration; providing nutrients—particularly for enterocytes	Reflux, small intestinal dysfunction (ileus, obstruction), esophageal trauma	Potential for stomach to rupture if small intestinal dysfunction and not properly monitored, epistaxis, esophageal trauma	$	Tolerance of nasogastric tube
PR	Unlikely to be of benefit	Appropriate choice	Appropriate choice	May have additional benefits for small colon impaction	Rectal tear	Limited reports suggest good safety	$	Tolerance of rectal catheter and rate Availability to stall

*IV, intravenous; IG, intragastric; PR, per rectum; GI, gastrointestinal*.

**Table 2:** Fluid therapy dosing recommendations for adult horses.

**Route**	**Indication**	**Fluid type**	**Dose**	**Comments**
IV	Resuscitation	Isotonic crystalloid	10–20 ml/kg bolus repeated as necessary to stabilize	Used in goal directed fashion with re-assessment of perfusion markers following each bolus. Caution with anuric/oliguric renal failure or heart disease.
		7.2% hypertonic saline	4 ml/kg bolus	Administration must be followed with isotonic crystalloids. Monitoring of electrolytes warranted with prolonged use.
		Hydroxyethyl starches	10 ml/kg/day	Higher rates associated with higher risk of coagulation derangements. Cannot monitor response with TP assessment due to falsely low reading on refractometer.
		Plasma	[(TPdesired – TPpatient)/TPdonor] ×0.05 BW	Monitor for transfusion reactions.
	Maintenance	Isotonic crystalloid	40–60 ml/kg/day	This requirement likely reduced in an adult horse off-feed, although exact requirements in these cases unknown.
IG	Maintenance	Plain water or Electrolyte solution	40–60 ml/kg/day Administered as 4–6 L every 4–6 h or as a constant rate infusion	Typical electrolyte solution recipe: 5.27 g NaCl (table salt), 0.37 g KCl (lite salt), and 3.78 g NaHCO3 in 1 L water. Volumes as high as 8–10 L per bolus reportedly well-tolerated. Volumes over twice maintenance for ingesta hydration not of any additional benefit.
	Fecal hydration	Plain water or Electrolyte solution	Up to 2 × maintenance (i.e., 80–120 ml/kg/day) Administered as 4–8 L every 2–4 h or as a constant rate infusion.	
PR	Maintenance	Plain water	5 ml/kg/h *via* constant rate infusion	Plain water reportedly better tolerated than electrolyte solution. Bolus dosing may be tolerated but has not been evaluated in the literature.

*IV, intravenous; IG, intragastric; PR, per rectum; GI, gastrointestinal; TP, total protein; PCV, packed cell volume; BW, body weight in kg*.

### Medical Indication for Fluid Administration

An important first question when choosing the route of administration should be: what is the purpose of fluid therapy? The indications for fluid administration are diverse, but can be generally categorized into emergency resuscitation, replacement of deficits, meeting daily requirements, and targeted treatment of a specific problem. Emergency fluid resuscitation is needed for shock due to decreased effective circulating volume (preload) and circulatory collapse associated with conditions such as severe acute hemorrhage or sepsis. Fluids are also indicated to replace existing deficits, or dehydration. Daily fluid requirements include both maintenance administration to cover expected sensible and insensible losses and to offset ongoing losses. Specific reasons for fluid therapy may include providing nutrients, correcting acid-base and electrolyte abnormalities, diuresis, and, particularly in horses, increasing water content in the ingesta and feces. Although for some indications the choice of route is clear, as is the case for most treatment decisions, more often it is not a black and white or right and wrong answer. For example, IV fluids are a clear choice for resuscitation, but correction of dehydration can be achieved *via* many routes.

#### Resuscitation

One of the most common reasons for fluid administration is resuscitation of the critically ill patient with insufficient preload due to hypovolemic, distributive, or obstructive shock. These patients require rapid, significant intravascular volume expansion. It should not be surprising that this is best achieved by administering fluids directly into the vascular space (IV). IG and PR routes require absorption and the amount that can be administered as a bolus or over a short period of time is limited. Additionally, in the hypovolemic state, perfusion to the gastrointestinal tract is reduced which slows absorption, further restricting the use of enteral routes for stabilization. Thus, the IV route is the clearly superior method for initial therapy. Once the intravascular volume has been sufficiently expanded and perfusion to the gastrointestinal tract is restored, replacement of deficits with IG or PR may be effective.

Commonly, fluid resuscitation in the equine patient is performed using large volumes of IV isotonic crystalloids, administered as a bolus. Recommendations regarding total dose have varied, although most focus on administration of boluses of approximately 20–25% of a total “shock dose” (80–90 ml/kg for horses) repeated (up to 4–5 times) until indices of perfusion have improved (10). In the horse, the current approach is typically to administer a 10–20 ml/kg bolus of crystalloid followed by reassessment of indicators of perfusion (e.g., heart rate, capillary refill time, pulse quality, extremity temperature, systemic lactate, urination, blood pressure, etc.,), and intravascular volume (jugular fill) (11). In the average 500 kg horse, this bolus would consist of 5–10 L of fluids, which if to be administered rapidly, requires a large (10–14) gauge catheter, wide-bore administration set, and the ability to suspend the fluids from a sufficient height above the horse (10). Alternative methods of volume expansion include administration of hypertonic solutions and/or colloids, as discussed in more detail in **Intravenous Fluids - Crystalloids** and **Intravenous Fluids - Colloids**.

#### Dehydration

Dehydration is a loss of total body water. In general, more fluid is lost from the extracellular fluid space, and the body preferentially takes fluid from the interstitial space to preserve the circulating volume. In horses, the third space of the gastrointestinal tract serves as a large reservoir of body water accounting for ~20% of total body water (12, 13). If losses become severe enough, hypovolemia can occur with dehydration and resuscitation would be required. But, if the intravascular volume is successfully spared, correction of the fluid deficit is not an emergency. Thus, treatment can be slower and *via* a variety of routes. In fact, IG and PR routes have been shown to be at least as effective as IV routes for correcting dehydration (14, 15).

Fluid deficits associated with dehydration are estimated based on percent dehydration. Typically, physical exam parameters such as mucous membrane moisture, skin turgor, and relative position of the globe are used when estimating the degree of dehydration, however many of these are unreliable in the horse (16–19). As percent dehydration increases, physical examination findings that are recommended as indicators begin to include changes consistent with decreased intravascular volume, poor perfusion, and shock such as prolonged capillary refill time, tachycardia, tachypnea, cold extremities, decreased mentation, and poor pulse quality (16). Other clinical indicators of dehydration might include loss of body weight, increased hematocrit and total protein concentrations, or electrolyte and serum osmolarity elevations (17). Bioelectrical impedance has been shown to provide accurate assessment of acute fluid shifts, but is not used clinically (20).

While many publications site specific estimates of dehydration and the expected associated changes in clinical parameters, it is likely that many of these are overestimates based on clinical findings during experimental induction of dehydration in horses. When water is withheld to induce dehydration, variable results have been reported. An older study evaluating 72 h of water restriction resulted in an average of 10.7% dehydration, while 24 h of restriction has resulted in a 3–6.3% decrease in body weight in other studies (14, 21, 22). Although horses in those studies did not experience signs of shock, other studies performed to evaluate the cardiovascular effects of dehydration reported two thirds of horses who were dehydrated to the point of 6.5–7% developed severe neurologic signs, signs of colic, and significant hyperlactatemia consistent with shock (23, 24). These findings suggest that severe dehydration would develop more slowly than duration of disease in many cases presenting with critical illness, and that percent dehydration traditionally considered as moderate can result in severe clinical signs in some horses. The likely overestimation of dehydration based on clinical parameters may be related to the inclusion of parameters associated with shock at higher percentages. It is important to consider that, although dehydration is one potential cause for hypovolemia, diseases associated with critical illness in horses can result in decreased preload through a variety of mechanisms. Thus, these findings are not specific for dehydration and may result in overestimation of dehydration if the underlying disease is more complex.

#### Daily Requirements

Fluid therapy plans must also account for the daily needs of a patient. Daily fluid requirements for all patients include those required for maintenance, as well as those needed to account for any ongoing losses. All animals have a basal maintenance fluid requirement that must be met in order to cover expected sensible (urine and fecal) as well as insensible (sweat and respiratory) losses. In the adult equid, this requirement is typically reported to be 2–4 ml/kg/h or 40–60 ml/kg/day, although recent work suggests a significant reduction in this requirement when horses are off feed (16, 25). As these maintenance requirements do not necessitate rapid fluid administration, they can be provided *via* routes other than IV, where no contraindications exist. In addition to these needs, any patient that exhibits ongoing losses, such as nasogastric reflux, diarrhea, or third-space losses, will require these losses be replaced in order to maintain normal fluid balance. In some cases, the extent and rapidity of these losses necessitates IV therapy, whereas, in other cases some or all of these losses can be met *via* alternative routes. Importantly, regardless of the route used, all types fluid intake must be considered when making treatment plans – particularly when an animal may be receiving medications, nutritional supplementation, and blood products in addition to their specific fluid therapy.

Specific problems that are treated with fluid therapy include hydration of intestinal contents; provision of nutrition; correction of anemia; treatment of failure of passive transfer; or reduction of cerebral edema, to name a few. Logically, the type and route of fluid therapy required will vary based on these underlying problems. For example, when considering the aforementioned conditions, the practitioner may utilize: IG fluid administration for improving ingesta/fecal water content; parenteral (IV) or enteral (IG) routes to meet nutritional needs; IV administration of whole blood; IV administration of hyperimmune plasma for failure of passive transfer and treatment of specific conditions (e.g., *Rhodococcus equi*, colitis, and coagulopathies); or IV administration of a hypertonic crystalloid or mannitol for cerebral edema.

### Medical Contraindications for Fluid Administration

Just as there are specific indications for particular types and routes of fluid administration, there are also specific contraindications. For example, the presence of nasogastric reflux precludes the use of IG fluid therapy, while patients in which venous access cannot be achieved may only be treated *via* enteral routes, and in horses with frequent diarrhea PR fluid therapy may not be worthwhile. The presence of cardiac disease and renal disease can also present challenges in developing a fluid plan. One good example is the patient suffering from cardiac failure who is showing signs of shock from poor perfusion and/or pulmonary edema. Although an IV bolus of fluids would be the most appropriate therapy for other types of shock resulting in poor perfusion, horses with cardiac failure are typically euvolemic or even hypervolemic and would be harmed by large volume resuscitation efforts. In horses with renal disease, it is important to differentiate anuric and oliguric from polyuric disease. While it is easy to volume overload patients that are anuric or oliguric, patients that are polyuric have higher maintenance requirements. Renal disease can also alter the ability to actively remove or retain electrolytes leading to the potential for alterations in sodium, potassium, and calcium, among others.

### Safety

As with any other prescription drug, safety is an important consideration when prescribing fluid therapy. Fluid therapy safety issues can be largely divided into those associated with technical and physical aspects of its administration and those associated with incorrect drug choice (fluid type or additives) or dosing (rate of administration).

While IV fluid administration is commonplace, it is perhaps the route posing the highest risk for complications – due to both the requirement for an indwelling IV catheter and the potential for medical errors when selecting and tailoring the fluids themselves. Catheter complications can vary in severity and consequence and may include phlebitis or thrombosis, septic thrombophlebitis, insertion site abscessation, or acquisition of an air embolus (26). The administration of fluids directly IV means that mistakes in fluid therapy calculations and/or supplementation are likely to be associated with more severe effects, as their uptake cannot be regulated by the intestinal tract in the way that enterally administered fluids might be. Fluid overload is a concept that has, to date, been largely ignored in our adult large animal patients, due to a perceived low risk. However, there has been a recent surge in interest and concern regarding the implications fluid overload might have on disease process and outcomes, particularly related to cases where signs of overload are less obvious. Fluid overload is discussed further in **Rate/Dose of Crystalloid Administration**.

Given the concerns regarding complications associated with IV use in both humans and animal patients, enteral administration has been investigated as a safer route that may also be associated with particular benefits. However, both IG and PR administration are associated with their own potential complications. IG administration requires either repeated passage of a nasogastric tube or the maintenance of an indwelling tube – both of which can be problematic. Repeated passage of a nasogastric tube is often poorly tolerated by the horse and increases the risk for trauma to the nasal passages, ethmoids, and pharynx, and also eliminates the possibility for continuous administration. Maintenance of an indwelling tube, however, can result in pharyngitis, laryngitis, and sinusitis, while reducing the ability to offer free water and feed to horses that might otherwise tolerate it (27, 28). Smaller diameter, softer indwelling tubes may reduce some of these concerns, but are generally more difficult to place and can be challenging to maintain in the authors' experience. Additionally, when administering fluids IG, the practioner must be mindful of the limits of the GI tract. Administration of fluid *via* an IG tube must not exceed the stomach capacity of the horse and, as noted previously, requires a functional proximal gastrointestinal tract. Mixing errors when preparing electrolyte solutions for administration can result in electrolyte and acid base abnormalities and exceeding recommended dosing for MgSO_4_ as a laxative can result signs of Mg toxicity. While little objective evaluation of the PR administration route exists in horses, some concern has been raised about the type of fluid administered. It has been suggested that hypotonic solutions, such as tap water, may be damaging to the rectal mucosa (29). However, a pilot study performed as part of an experimental study evaluating PR fluid administration found that horses tolerated tap water better than a more isotonic polyionic solution (29). While the reported method of administration *via* a small, soft indwelling catheter is apparently well-tolerated, and considered unlikely to cause significant trauma to the rectum, this cannot be said about other administration devices.

### Cost

Sterile, commercially available fluids meant for IV administration in horses, and the materials needed to deliver them, are expensive. While cost varies significantly by location, in the authors' experience, it is not uncommon for the catheter, administration sets, and fluids necessary to administer even just a shock bolus of fluids to be in the realm of hundreds of dollars. In addition to the cost of the actual materials, IV fluid administration requires close monitoring by trained individuals. While some practitioners may feel comfortable with certain clients assuming the risks, this typically requires practitioners to either stay in the field for the duration of therapy, or, more ideally, requires hospitalization – each of which associated with significant cost to the client. In both human and veterinary patients, the move toward enteral fluid therapy has been sought not only because of a perceived improved safety margin and particular benefits for specific problems, but also in large part due to a reduction in workload and cost (30, 31).

In an attempt to reduce the cost of IV fluid therapy in cases where it is warranted over enteral routes, the use of non-sterile, homemade fluids (colloquially also known as “jugs”) has been an alternative to commercially available options. These fluids are generally formulated by adding a powdered electrolyte mixture to commercially available reverse osmosis or distilled water meant for human consumption (32). Non-sterile fluids have generally been restricted for use in budget cases or when medical grade fluids have been be in short supply. When commercial alternatives are not available due to shortages, this option seems justified. However, when the only impetus for their use is on the basis of cost, the justification is less clear and legal implications of administering compounded drugs over FDA approved and regulated drugs should be considered. Commercially available fluids are produced in appropriate clean environments and guaranteed to be non-pyrogenic and endotoxin-free, features that cannot be achieved during homemade mixing of potable water, regardless of the source. A recent study compared bacterial and endotoxin contamination in hand mixed non-sterile IV fluids made from chlorinated drinking water and chemical grade electrolytes, compounded IV fluids made from distilled, filtered, irradiated water and filtered electrolyte solution and subsequently autoclaved, and commercially available balanced polyionic fluids (Plasma-Lyte A^1^) (33). This study showed a significant increase and unacceptable level of bacterial contamination in hand-mixed fluids (7/8) compared to compounded (1/8) and commercial (0/8) fluids. The study also detected a low level of endotoxin in 1/8 of the hand-mixed samples and no samples from the other two fluid types. An earlier, small study had suggested significant risk of endotoxin contamination resulting in clinical signs of endotoxemia in non-sterile fluids made using reverse osmosis water and stored in plastic containers (34). A recent retrospective comparing outcomes between horses treated with commercial IV fluids vs. homemade solutions reported no differences in survival to discharge (32). However, the horses treated with homemade fluids reportedly developed more jugular vein complications and hyperchloremic metabolic acidosis while in hospital (32). A similar increased risk for jugular vein complications has been reported in association with non-sterile fluid use in a previous study (35). The hyperchloremic acidosis seen in the study was unsurprising, given the high chloride content (152–153 mmol/L) of all non-sterile fluid formulations (32). The authors of this study described an alternate formulation where some of the potassium was provided as potassium bicarbonate as opposed to potassium chloride, however, the use of this formulation has yet to be evaluated (32).

### Patient and Environmental Factors

In addition to the considerations discussed above, sometimes circumstances limit the options available to a patient. Patient temperament can affect the choice of fluid administration route. For example, horses have variable tolerance of indwelling nasogastric tubes and some have a propensity for removing their own catheters. While there are ways to mitigate these issues, if other factors are equal this should be a factor in planning. Where and how the horse is housed can also affect the choice of route of fluid administration. For example, constant rate fluid administration may not be an option in extreme cold due to freezing and rapid IV administration *via* gravity flow requires fluids to be suspended at a significant height. It should be noted that, if facility limitations are likely to negatively impact the standard of care the patient receives, then hospitalization should be considered.

## Intragastric Fluids

IG therapy has a long history of use in equine fluid therapy, with proposed benefits over IV administration that include reduced cost, ease of administration, and improved safety. In addition to these considerations, there is some evidence to suggest that IG administration may be preferable to IV under some circumstances.

### Indications for IG Administration

IG fluid therapy should be considered as a route of fluid administration for replacement of dehydration, providing maintenance requirements, and treating specific diseases. While IG administration is a becoming a more commonly utilized route for treating horses suffering from large intestinal impaction, for other equine cases it is frequently overlooked and has been the subject of little research. IG administration of either isotonic electrolyte solution or plain water has been shown to increase fecal water content more effectively than IV fluid therapy (14, 36). Treatment with plain water was shown to result in a rate-dependent improvement in fecal water content when administered at a maintenance fluid rate (50 ml/kg/day), twice times maintenance (100 ml/kg/day), or three times maintenance (150 ml/kg/day) (14). A previous study has reported increased efficacy using balanced electrolyte solution rather than plain water IG or IV fluid administration for right dorsal colon ingesta hydration (36). Lester et al. (14) found that IG fluid therapy in dehydrated horses had a rate-dependent effect on serum osmolality and sodium concentrations, suggesting systemic absorption. These benefits were actually above and beyond those seen in horses administered IV fluids, implying that IG may be a very effective route for rehydration (14). Two retrospective studies have reported positive outcomes in treatment of large colon impactions with IG fluid administration (37, 38).

Similar benefits of oral rehydration therapy have been documented in humans. In children, oral rehydration has become the recommended first line therapy for mild and moderate dehydration (30, 31, 39). Initial studies in horses evaluating rehydration after exercise supported the use of oral rehydration solutions for restoration of circulating volume after exercise (40, 41). These studies evaluated several indirect indices of circulating and total body water volume (i.e., plasma volume, changes in total protein, body weight) following rehydration with plain water or an isotonic oral rehydration solution *via* nasogastric tube (40, 41). They reported evidence of changes in circulating volume despite small volume (4 L) administration, and that an isotonic oral rehydration solution appeared more efficacious than water, although both appeared safe (40, 41).

Interestingly, despite the evidence, there continues to be a tendency to rely on IV fluid therapy in people and equine practice based on similar arguments: perceived ease of use and more rapid patient response, as well as the expectations of clients and referring clinicians (39). The authors suggest that, provided the horse has a functional proximal intestinal tract, evidence supports IG fluid administration as an easy, inexpensive route of administration for systemic and gastrointestinal hydration.

### Types of Fluid for IG Administration

The most appropriate solution for IG administration remains a topic of debate. In human medicine, there is a general acceptance that an electrolyte rehydration solution, rather than plain water, should be utilized and, in almost all cases, these solutions include a glucose or dextrose source (30, 31, 39). Additionally, most human formulations also contain some buffer component – most commonly bicarbonate or citrate.

The theory behind using an electrolyte solution rather than water relates to the net effect this will have with respect to subsequent fluid shifts. When plain water is administered, it will be absorbed and equally divided between the extracellular fluid space (one fourth of which is in the intravascular space). The extracellular fluids will then be relatively hypotonic and further redistribution into the intracellular fluid space will occur to balance tonicity between these compartments. By comparison, if an isotonic electrolyte solution is administered, the fluid will once again be initially taken up and equally divided between the extracellular fluid spaces. However, there will not be the osmotic drive to initiate further redistribution intracellularly, and therefore more of the absorbed fluid will remain in the extracellular (and, thus, intravascular) space. Although plain water and electrolyte solutions increase gastrointestinal water content and plain water has been shown to correct dehydration in horses, concerns have been raised regarding the hypotonicity of plain water resulting in the possibility of serious electrolyte abnormalities (36). However, no clinical signs were reported in relation to the mild hyponatremia in a one study, and a second investigating the use of plain water reported no complications with its use (14, 36). Evaluation of horses administered furosemide and then exercised to induce dehydration found that consumption of salt water resulted in more fluid intake and body weight gain in the first 1 h compared to plain water (42).

The purpose of the glucose/dextrose component in human rehydration solutions is to drive glucose cotransport of sodium within the intestinal tract, ultimately creating more solvent drag and fluid uptake. Interestingly, however, several studies in horses have not supported their use over a electrolyte solution not containing glucose (43, 44). Ecke et al. (45) investigated the use of a glucose -based rehydrating solution commonly used in diarrheic calves for treatment of adult horses with experimentally induced diarrhea. They concluded that it was not effective for this purpose, and in fact precipitated acid-base and electrolyte derangements (45). More recently there has been a shift toward polymer-based rehydration solutions that contain starch components rather than glucose or dextrose. The aim of these solutions is a slower, more sustained glucose release and less abrupt increases in intra-luminal glucose, which may actually act counterproductively by creating an osmotic drag into the gastrointestinal lumen and net loss of fluids (31). To the authors' knowledge, such polymer-based oral rehydration solutions have not been used in equine patients.

The buffering component of human rehydration solutions is included in an attempt to counteract metabolic acidosis, particularly in diarrheic patients. Many of the oral rehydration solutions used in the horses do include sodium bicarbonate in varying proportions (e.g., 5.27 g NaCl (table salt), 0.37 g KCl (lite salt), and 3.78 g NaHCO3 in 1 L tap water) (16). However, a comparison of sodium chloride solution to a more complete buffered electrolyte solution has not been performed in studies of experimental dehydration to the authors' knowledge. The authors are also unaware of any studies evaluating the use of buffered solutions for either voluntary consumption or IG administration in horses with diarrhea, although some clinicians will provide horses with the choice of electrolyte water clinically.

### Methods and Rates of IG Fluid Administration

Intragastric fluids can be administered either as a bolus or as a continuous infusion *via* nasogastric tube. Bolus dosing can be convenient, as it does not require a stall set-up that allows for a continuous administration. With gastric emptying times for fluid reported at around 15 min, frequent boluses of appropriate volumes should be possible and well-tolerated, so long as normal small intestinal function is adequate (46). However, there is a limit to how much can be administered at any one time given the capacity of the stomach, the maximum of which has been reported to be approximately 15–18 L in the average adult horse (47). Interestingly, Lester et al. (14) were able to administer up to three-times maintenance over four treatments, which, based on the weights in their population would be as much as almost 20 L at each interval. Despite administering more than the reported capacity of the stomach, they reported being able to administer this over 15 min with no ill effects (14). Monreal et al. (37) similarly describe administration of volumes as high as 8–10 L every 2 h, with no signs of discomfort attributed to this treatment. In the authors' experience, it is better tolerated to administer smaller volumes more frequently, such as 4–6 L of fluids every 2–4 h. If administering as a constant rate infusion, a rate of 1–2 L per hour has been recommended (15).

## Rectal Fluids

Rectal fluid administration has gained recent interest within equine practice as an attractive alternative because of the ability to administer relatively large volumes with very little cost. Use of this route precludes the requirement for sterile fluids, and the need for precise tailoring of the fluid balance. While rectal fluid therapy has been reported in other species, including humans, studies in horses have been limited to date (48–52).

### Types and Rates of Fluid Administration PR

The first study the authors can identify evaluating PR fluids in horses was performed in 1979 evaluating administration of saline PR. In that study, 44 L of saline were administered to horses with furosemide-induced dehydration (53). An acidifying effect of the saline was observed (see **Balanced Polyionic vs. Saline** for more on the potential detrimental effects of saline), but no effect on hydration was identified. A separate part of this study evaluated how far orally fluid migrated in horses and found it rarely reached the level of the pelvic flexure (53). More recently, a letter to the Veterinary Record reports briefly on one practitioner's use of PR fluid administration and two studies have been performed in horses evaluating PR fluids - one small study in horses with naturally occurring mild dehydration and another experimental study comparing IV, IG, and PR fluids in healthy horses (15, 29, 54).

A 2018 study evaluated PR therapy in eight horses with naturally occurring mild dehydration (6% estimate based on clinical signs). Horses that were deemed to be mildly dehydrated, based on loss of body weight and evidence of hemoconcentration, were administered their calculated fluid deficit of a homemade polyionic solution PR in boluses of no more than 5 L at a time. The procedure was well-tolerated and resulted in a measurable improvement in hematocrit and total protein concentrations, with minimal changes in overall electrolyte balance (15).

A 2019 study evaluated both the tolerance of equine patients to rectally administered fluids, as well as the resulting effect on clinical chemistry changes in an effort to confirm absorption (29). An initial pilot study was performed in order to determine the most appropriate type (polyionic solution or plain tap water) and rate of administration (2.5, 5, 7.5, and 10 ml/kg/h) of fluid to be used in the main study. Due to less tolerance of polyionic fluids and higher rates during the pilot study, the horses were subsequently administered plain tap water at 5 ml/kg/h (roughly 2–2.5 times maintenance rate) *via* continuous gravity flow over the course of a 6-h period. In the main randomized, crossover study, six horses were treated with each of 3 fluid therapy treatments (polyionic solution IV, polyionic solution IG, or water PR at a rate of 5 ml/kg/h) and a control treatment where no fluid was administered. Results indicated that horses administered tap water per rectum at these rates tolerated this well and exhibited evidence of hemodilution (based on decreased PCV/TS) similar to that achieved *via* the IV and IG routes (29). The authors concluded this to be both a safe and effective means by which to either replace or augment other routes of fluid administration. It should be noted, however, that the effects of administering hypotonic tap water on the rectal mucosa was not evaluated.

### Methods for PR Fluid Administration

In the more recent equine reports, PR fluids have been administered *via* gravity flow through either a small, well-lubricated esophageal tube passed 10–15 cm into the rectum or a small (24 Fr) soft enema tube (15, 29, 54). In the authors' experience, placement of a long foley catheter or red rubber catheter has been well-tolerated. Placement of a single interrupted suture between the edge of the catheter and the anus or perineum has been beneficial in keeping the catheter from falling to the ground if displaced, as has been seen to occur with higher fluid rates or defecation.

## Intravenous Fluids - Crystalloids

Crystalloid fluids are the mainstay of IV fluid therapy, both in human and veterinary medicine. Much debate has developed with respect to the best type and rate of crystalloid administration.

### Types of Crystalloids

Crystalloids can be defined based on their tonicity, use, and/or electrolyte composition. Most crystalloids are isotonic, meaning that they have a similar tonicity to fluid within the body, both intra- and extra-cellularly. Sodium is the main determinant of solute concentration and, therefore, osmolarity in extracellular fluids within the body and in IV fluids. Thus, low sodium fluids are hypotonic unless a solute, usually dextrose, is added and high sodium fluids are hypertonic. Most commonly, low sodium fluids fall into the category of fluids used to maintain total body water (fulfill daily fluid requirements). High sodium, hypertonic solutions, are most frequently utilized to rapidly shift fluids into the intravascular space during resuscitation and can also be useful for treatment of cerebral edema. Correction of sodium imbalance is another indication for administration of hypo- or hyper-tonic (low or high sodium) fluids. The distinctions between most commonly available commercial crystalloid solutions are discussed below. In addition to these basic crystalloid options, some additional alternatives exist, such lactate-rich polyionic solution and bicarbonate solutions, which have specific purposes related to restoring circulating volume or normalizing acid-base disturbances (55, 56). Modification of the common commercial solutions with additives is also commonplace to address electrolyte and acid-base imbalance and supplement nutrients.

#### Replacement vs. Maintenance

Replacement fluids are designed to “replace” deficits, generally those associated with hypovolemia and dehydration. Maintenance fluids are designed to “maintain” homeostasis by providing requirements associated with maintenance needs and expected losses. Because the deficits being replaced in cases of hypovolemia (intravascular) and dehydration (preferentially interstitial) involve the extracellular fluid space primarily, replacement fluids have a composition similar to extracellular fluid—high in sodium and chloride, low in potassium, calcium, and magnesium. In contrast, the losses being addressed with maintenance fluids are lost from both intra- and extra-cellular fluid spaces through fluids such as urine, which are generally comparatively low in sodium and chloride and high in potassium, calcium, and magnesium. Thus, maintenance fluid composition is closer to total body water electrolyte composition. The low sodium concentration of maintenance fluids makes them hypotonic unless dextrose is added.

Commercially available replacement fluid options available include: 0.9% sodium chloride (0.9% NaCl)^2^, lactated Ringer's solution (LRS)^3^, Normosol-R^4^, Plasma-lyte A^1^, and Plasma-lyte 148^5^ (11). Commercially available maintenance options include: Normosol-M^6^, Plasma-lyte 56^7^, 2.5% dextrose in half-strength saline (0.45% NaCl + 2.5% dextrose)^8^, and 5% dextrose in water (D5W)^9^ (17). While clearly a variety of options exist, the reality is that most clinics only stock one and at most a few options in sizes appropriate for use in adult horses. When a single fluid is stocked, in general it is a replacement fluid, as these are available in large sizes, are most commonly used, and can be given rapidly to patients in need of resuscitation. However, given the electrochemical makeup of these fluids, maintaining patients on this type of fluids, beyond the initial replacement period, inevitably results in sodium loading and inadequate replacement of other electrolytes. While potassium, magnesium, and calcium can be supplemented in replacement fluids, without dilution of the base fluid, the sodium load remains an issue.

Such a significant sodium load results in natriuresis and thus diuresis, which is logically counterproductive and can result in depletion of additional electrolytes. In the study by Lester et al., kaliuresis and diuresis persisted in the recovery period following administration of IV polyionic replacement fluids. Horses in the IV treatment group were unable to completely restore hydration during the treatment period and illustrated a rebound dehydration during the 24 h following treatment (14). Similar incomplete rehydration was reported in an earlier study using saline for rehydration in horses (57).

Sodium that is not able to be excreted contributes to fluid retention and predisposes to fluid overload and development of edema. The general approach to fluid therapy in adult equids has been to consider them relatively tolerant of overzealous fluid therapy efforts in comparison to their small animal counterparts, due in large part to the comparatively fewer cases of cardiac dysfunction or renal insufficiency. However, in the diseased adult patient or neonate with less tolerance for large sodium shifts, the potential for sodium and fluid overload should be acknowledged and avoided.

#### Balanced Polyionic vs. Saline

A recent controversy within crystalloid fluid therapy is the shift away from using 0.9% NaCl as the replacement fluid of choice in favor of a more balanced electrolyte solution. Historically, saline has been the mainstay fluid of choice in human medicine due to the perception that it is the most physiologically appropriate choice. However, this has been questioned due to concerns regarding the effects of increased chloride load from its administration (58–60).

Physiologic 0.9% saline, or normal saline, contains sodium and chloride only, and each of these components exists in higher levels in this fluid than is present in normal equine plasma (154 vs. ~130–140 mmol/L for sodium and 154 vs. ~90–100 mmol/L for chloride) (11). Normal saline administration thus results in a high sodium burden, which stimulates natriuresis which may be counterproductive in patients suffering from hypovolemia or dehydration. A potentially larger concern regarding the use of normal saline, however, is associated with the high chloride burden following administration. This has been shown to consistently result in a hyperchloremic metabolic acidosis – hence the common description of saline as an “acidifying fluid.” This effect on acid-base balance can be explained in the context of strong ion difference, as the increase in chloride load results in a decreased strong ion difference, a decrease in bicarbonate and base excess as a result, and ultimately a reduction in blood pH. In patients already suffering from acid-base disturbances, this may be particularly contraindicated. A study in endurance horses comparing saline to acetated polyionic fluids showed a decrease in sodium-chloride difference consistent with the potential for a hyperchloremic metabolic acidosis in horses administered saline (60). Furthermore, saline use in both healthy and critically ill humans has been shown to result in an increased risk for renal compromise, thought to be explained by a chloride-mediated renal vasoconstriction and subsequent reduction in renal perfusion (58, 59, 61). Despite these concerns, a large Cochrane review evaluating 21 randomized controlled studies and three ongoing studies was unable to identify an improved mortality in human critically ill adults or children treated with a buffered solution vs. normal saline (62).

While a balanced electrolyte solution is favored for the majority of equine patients, there are certain disease states in which sodium chloride would be preferable. The first of these is logically patients with a hypochloremic metabolic alkalosis, as the propensity for a hyperchloremic metabolic acidosis may actually be of benefit in correcting this disturbance. Normal saline has also generally been selected preferentially over balanced electrolyte solutions in hyperkalemic patients. This is because all balanced electrolyte solutions contain some potassium, which may theoretically worsen the hyperkalemia. However, the acidosis induced by high chloride fluids induces a shift of potassium out of cells which may increase potassium to a greater degree than lactated ringer's solution (63, 64). Lastly, sodium chloride is considered superior in cases of head trauma, as it is thought that the relative hypotonicity of balanced electrolyte solutions may result in fluid shifts that could result in increased intracranial pressure (59).

#### Hypertonic Saline

Most commonly hypertonic saline is available as a 7.2% solution^10^, with sodium content of 1,232 mOsm/L, resulting in almost nine times the tonicity of plasma (65). Administration of this hypertonic solution results in significant and rapid fluid shifts into the intravascular space, initially from the other extracellular compartments (i.e., interstitial space) and will continue from the intracellular space. As a result of these fluid shifts, the effective expansion of circulating volume is in the order of 3.5 times the administered volume (11). Despite such a rapid initial expansion, this effect is relatively short lived, due to redistribution of electrolytes and water across the vessel wall as expected with all IV crystalloid administration. Thus, for a sustained effect and, to avoid ill-effects of intracellular dehydration, hypertonic saline administration should be followed by larger quantities of IV isotonic replacement crystalloids. Administration of enteral fluids may provide an alternative to IV isotonic replacement crystalloids following hypertonic saline resuscitation. In calves with diarrhea and moderate dehydration and acidosis, small volume IV hypertonic saline followed with oral electrolyte solution was effective at rapidly improving hydration and acid-base status (66).

The rapid volume expansion achieved with hypertonic saline administration makes this an excellent choice in the resuscitation of the hypovolemic patient. In an experimental study in which anesthetized horses underwent controlled exsanguination to achieve induced hemorrhagic shock, administration of hypertonic saline resulted in rapid plasma volume expansion and urine output, along with sustained elevations in numerous cardiovascular parameters including cardiac output, stroke volume, mean arterial pressure, and contractility (67). Comparatively, administration of an isotonic saline solution only resulted in transient increases in mean arterial pressure but no changes in other cardiac parameters, plasma volume, or urine output (67). These rapid fluid shifts are also of benefit in patients suffering from life-threatening increased intracranial pressure and/or cerebral edema, as a means of reducing the intracranial volume. Other purported benefits of hypertonic saline include anti-inflammatory, anti-edema, and potential inotropic effects, although the last of these is difficult to separate from its volume expanding effects (11). Obvious contraindications exist, such as the already severely hypernatremic patient (unless the need to treat of cerebral edema supersedes this), as well as in patients with uncontrolled blood loss where the increase in circulating volume and inotropic benefits may actually be problematic.

### Rate/Dose of Crystalloid Administration

The rate of crystalloid administration is typically dictated by the indication for the fluid therapy. However, as briefly discussed above, to date there has often been an inclination to treat with very aggressive fluid therapy rates, especially for particular problems such as intestinal impactions. In addition to the concerns regarding high fluid rates resulting in counterproductive diuresis (particularly with high sodium content of replacement fluids commonly used in equine practice), other potential drawbacks of this approach include cost, lack of actual benefit, and the potential for detrimental fluid overload.

Interestingly, studies evaluating the use of IV fluids to improve fecal water content have been conflicting. Lester et al. (14) identified that rates of 100–150 ml/kg/day (~2–3 L/h or 2–3 times maintenance) did increase fecal water content, although there was no additional benefit of the higher rate over the lower. In contrast to this, Lopes et al. (36) found that rates as high as 5 L/h had no effect on the hydration of colon contents or feces. The difference in the results of these two studies may be related to the hydration status of the horses and, therefore their gastrointestinal contents, when fluids were administered. This suggests that extrapolation to clinical patients with highly variable diseases is unlikely to be straightforward and further investigation is needed. However, while IV fluid therapy may be of benefit in gastrointestinal hydration, the concept of aggressive “overhydration” is not supported by current literature and the effects of combining enteral and IV therapy is unknown.

As noted, the concept of fluid overload, a commonly considered issue in human and small animal medicine, has been largely ignored in treating the adult equine. Although, few adult horses suffer from cardiac or chronic renal disease predisposing to fluid overload, other risk factors of hypoproteinemia and systemic inflammation are frequently found in the critically ill equid. Additionally, many of these same patients are at risk for acute kidney injury and compromised renal function. In human medicine, aggressive fluid therapy in both the perioperative and intensive care settings has been associated with the development of fluid overload and subsequent ill-effects including interference with gas exchange due to the development of pulmonary edema, impaired wound healing, compromised renal function, and increased mortality in septic and post-operative patients (2, 68). As a result, there has been a shift toward a more tailored fluid therapy approach that is adjusted in relation to the patient's status and response and a focus on de-escalation, and even evacuation *via* diuretic therapy, when appropriate (2, 68). An additional consequence of fluid overload in people that is of particular interest to equine practitoners is the negative impact of fluid overload on gastrointestinal healing and post-operative function. It been theorized that aggressive peri-operative fluid therapy can result in subsequent gastrointestinal wall edema which may contribute to poor gastrointestinal function and post-operative reflux (POR) (69). Studies of restricted fluid therapy have shown this to be associated with a decreased risk of ileus and improved outcomes in human patients (70, 71). Further to this, electrolyte abnormalities, which can be induced by aggressive fluid therapy, are known to contribute to POR in other species, further supporting re-evaluation of the fluid approach in these patients (71, 72). One study evaluating the influence of both total fluid volume and electrolyte derangements on the incidence of POR in horses did not identify either of these as factors contributing to the development of POR (69). However, this was a single study that was retrospective in design and did not have a means for accurate and consistent measure of fluid balance. Furthermore, most fluid rates reported in this study would not have been classified as aggressive based on human medical standards, which are commonly on the order of 240–440 ml/kg/day, vs. the 102–117ml/kg/day reported here (69). Thus, further investigation is warranted. In the meantime, monitoring for fluid overload in all critical patients is probably indicated. While quantitative monitoring of “ins and outs” is difficult, monitoring for indirect signs such as significant weight gain or development of peripheral edema is easy to do. Central venous pressure as a method to evaluate intravascular volume has been evaluated in horses, but clinical use is difficult due to inconsistent results associated with technical errors and potential for effects of confounding diseases/conditions.

## Intravenous Fluids - Colloids

Colloids can be broadly grouped into two types: natural and synthetic. Natural colloids include whole blood, plasma, and albumin, while synthetic colloids include hydroxyethyl starch (HES) solutions, dextrans, and gelatins. Hetastarch^11^ is the most widely used and available HES, but other formulations including Pentastarch^12^ and Tetrastarch^13^ can be found and have been evaluated. The different formulations of HES solutions vary in molecular weight, substitutions, and carrier solution. While previously considered an important component of the stabilization and treatment of the critical care patient, colloids have seemingly fallen out of favor in both human and veterinary medicine. Much of the decrease in use is related to the controversies surrounding potential harmful effects, particularly in patients with critical illness, specifically sepsis and burns, and the resulting regulations in Europe related to the use of HES. This began in late 2013, when the European Medical Agency stated that HES should not be used for patients with sepsis, burns, or critical illness, in response to the results of several landmark studies showing increases in mortality and requirement for renal replacement therapy in septic patients (73–75). The recommendations did allow for continued use for the treatment of acute hemorrhagic shock if crystalloids were not considered sufficient, for no more than 24 h and with kidney monitoring. Further, study was recommended for elective surgery and trauma patients. In 2018, the committee voted to suspend HES from the European market due to evidence of continued use of HES off label in septic patients (76). The vote was fairly close (15–13) and there are a number of correspondences and editorials that are against the ban (77, 78). Those in favor of continued use point to positive results in some studies, lack of consistent support of negative effects in multiple studies, and the ongoing clinical studies evaluating safety for elective surgery (PHOENICS (NCT03278548)) and trauma patients (TETHYS (NCT03338218)) (79–83). There are also concerns for the potential to worsen outcomes in conditions that benefit from HES, particularly in regions where alternatives are limited.

### Colloid Osmotic Pressure and the Proposed Role of Colloids in Health and Disease

Colloids are relatively large, charged, osmotically active molecules. The amount of osmotic pressure created by colloids in a solution is measured as colloid osmotic pressure and also known as oncotic pressure. The number of molecules, rather than their size, is responsible for the osmotic pull. For this reason, in plasma, it is albumin, not globulin, that is the predominant molecule responsible for oncotic pressure, because there are more molecules of albumin. It is for a similar reason that smaller molecular weight HES solutions should provide more volume expansion that larger molecular weight HES. Due to the large size and charge of colloid molecules, normal vessels are not permeable to colloids, which is in contrast to other osmotically active molecules such as electrolytes and glucose. Whereas, electrolyte and glucose concentrations quickly equilibrate between the plasma and interstitial spaces, colloids remain within the vascular space, maintaining an osmotic gradient that encourages transcapillary movement from the interstitium to the intravascular space and keeps water in the vasculature, providing a more sustained intravascular volume expansion (84, 85).

The underlying principle behind the use of colloid therapy to produce sustained volume expansion was the standard Starling equation of transcapillary movement. In summary, the equation states that the direction and amount of transcapillary fluid movement is related to the balance of hydrostatic and colloid osmotic, or oncotic, pressures between the capillary and the interstitium and a factor representing the permeability of the vessel wall (K_f_). If applied to crystalloid administration, the addition of isotonic fluids within the vascular space results in an increased volume and, therefore, hydrostatic pressure along with dilution of colloid/protein and, therefore, a decrease in oncotic pressure within the vessel. Both changes in the capillary favor a net movement of fluid into the interstitium. If applied to colloid administration, the increase in volume still results in an increase in hydrostatic pressure, but the increase in colloid results in an increase in oncotic pressure within the vessel, pulling fluid into the vessel. The goal is to increase the oncotic pressure enough to offset and outweigh the increased hydrostatic pressure to favor net movement of fluid from the interstitium and into the intravascular space. Thus, theoretically colloid administration should result in a more significant volume expansion effect for a given volume administered, with less redistribution of the fluid to the interstitium, providing a more sustained expansion and minimizing the negative effects of accumulation of interstitial fluid.

While this is a fairly straightforward concept to understand, the accuracy of the Starling equation when applied clinically has been called into question, creating doubt about the expected response to colloid administration in critical patients (86, 87). Two main problems exist with this concept as it currently stands: there is evidence to suggest that the interstitial space and its fluid balance changes in disease states; and the lining of the microvasculature may not allow the movement of fluid from the interstitium back into the vasculature as once thought (86, 87). With respect to the first of these, it has been shown that inflammation and other pathology can result in negative pressure within the interstitium, promoting transcapillary movement of fluid into this space and subsequent edema formation (88). The thought is that an increase in the plasma oncotic pressure would not necessarily overcome the vacuum-like draw of fluid into the interstitium and thus fluid accumulation would result. Further compounding this, the endothelial glycocalyx, which is the region of the vessel thought to regulate the movement of fluid between the vasculature and the interstitium, is proposed to limit the reuptake of fluid from the interstitium back into the capillaries (86, 87). This would mean the proposed benefit of added volume expansion following colloid administration is unlikely. Additionally, any fluid lost to the negative space of the interstitium may be trapped, especially if leaky vessels (increased K_f_) allow colloids to accumulate, along with the water they attract (86, 87). Smaller colloid particles (lower molecular weight HES, for example) are more likely to leak out of even normal vessels.

### Indications for Use of Colloids

The indications for colloid administration relate to the proposed role of the colloid osmotic pressure exerted by colloids in the fluid balance between the intravascular and interstitial spaces. Overall, administration of colloids intravenously is generally performed with one of two goals: improving colloid oncotic pressure (COP) or inducing more rapid and sustained volume expansion than crystalloids during fluid resuscitation of critically ill patients (89). Related to these goals, colloid administration has been suggested for treatment of two main pathologies: conditions resulting in decreased colloid osmotic pressure, such as protein losing enteropathy, and conditions requiring rapid expansion of intravascular volume. In addition to these indications for colloids as a whole, natural colloid options such as whole blood or plasma are often administered for a particular purpose in certain cases – such as replenishing red cells, plasma proteins, and coagulation factors in the case of whole blood, or for anti-endotoxic or coagulation benefits in the case of plasma. As the focus of this review is not transfusion medicine, the authors direct the reader to available literature on the uses, purported benefits, and drawbacks of these products (3–5, 90–94).

Despite the theoretical benefits of colloid administration, results of clinical studies have failed to consistently show advantages over crystalloids in human medicine (84, 85). Initial clinical studies in people did report significantly improved outcomes in critically ill patients who received colloid-based volume resuscitation in comparison to crystalloids (84). However, many of these publications have been discredited, and this has subsequently muddied the water with respect to the meta-analyses in which they were included (84). It seems likely that consideration of the underlying disease is important when assessing both the benefit and risk of colloid administration. Patients with critical illnesses like sepsis and burns have shown minimal to no benefit and there is evidence of worsened outcomes and side effects when treated with colloids when compared to crystalloids (95). In contrast, patients with hypovolemic shock, trauma, and undergoing elective surgeries may experience benefits and even those at risk for kidney injury may not have an increased risk of renal complications (79–81, 95). While there is some support for their use in equine patients, there has been no strong evidence for an overall improvement in outcome.

In general, regardless of the type, colloid products are more costly than crystalloid alternatives, when compared on a volume basis (85). However, if increasing the COP results in maintenance of fluid within the vascular space and decreased redistribution of crystalloids to the interstitial space, the end result could be reduced edema formation and requirement for crystalloid administration. This could potentially have the effect of reducing overall cost.

#### Improved Colloid Oncotic Pressure

With respect to improved COP, administration of synthetic colloids to healthy horses and ponies in an experimental setting has been shown to result in an increase in oncotic pressure for as long as 24 h post-administration (89, 96). When comparing the improvement in COP following administration of a synthetic colloid (Hetastarch or HS) and plasma to healthy horses, McKenzie et al. (97) reported a similar increase, regardless of the colloid used. Interestingly, despite its frequent use in anesthetized horses in an attempt to counteract the decreased COP seen under anesthesia, the effects of synthetic colloids in these settings is variable (98–101). A study comparing the effects of HS to isotonic crystalloids on COP in healthy patients undergoing elective surgery found higher COP following the colloid administration (101). In contrast, another study performed in a similar population compared the effects of isotonic crystalloid to a combination of crystalloid with synthetic colloid (HS) and found no difference in COP between the groups (100). When evaluated in critically ill horses suffering from hypoproteinemia secondary to gastrointestinal disease, synthetic colloid administration was shown to result in a significantly increased oncotic pressure (102). While administration in this study resulted in an ~20% increase in COP, no horses were seen to have a return of COP to that measured in healthy horses (102). A more recent retrospective study comparing outcomes in horses with enterocolitis treated with plasma to those treated with a synthetic colloid (HS) reported significantly better outcomes in the plasma treated horses (80 vs. 27% survival) (103). It should be noted, however, that another retrospective study published the same year reported that horses who had received a plasma transfusion for treatment of typhlocolitis/colitis had a higher odds of dying than non-transfused horses (90). The retrospective nature of this study makes it impossible to identify whether this was due to more severe disease in the transfused patients, as well as the reasoning behind the use of plasma in each particular case (i.e., low total protein, coagulation concerns, etc.,), making the interpretation of these findings particularly challenging (90).

#### Use in Fluid Resuscitation

While the colloid literature in human medicine has focused a great deal on outcomes (albeit with much controversy over the legitimacy of the results), similar studies within the equine literature are sparse. Several studies have evaluated the effect of colloid administration on hematologic parameters that are considered indicative of hemodilution, supporting the premise that these products enhance the circulating fluid volume. Previous reports have indicated that both synthetic colloid and natural colloid options reduce hematocrit (HCT) or packed cell volume (PCV) and/or total protein (TP) or total solids (TS) concentrations in healthy horse (89, 97, 100, 101, 104). A report from Epstein et al. documented an increase in systemic blood pressure following administration of HES products, while another by Ohta et al. documented an increase in cardiac output (despite failing to identify an increase in blood pressure), further supporting their role in resuscitation (89, 105). However, in an experimental study of anesthetized horses given endotoxin, resuscitation with hypertonic saline and HES did not reverse the negative cardiovascular effects of endotoxemia (106). Albumin, a natural colloid commonly used in people and with some frequency in small animals, was recently evaluated for its volume expansion effects in horses (107). Administration of an equine albumin product resulted in a reduction in HCT and TP, improved mean arterial pressure, and shorter capillary refill time (107). Clearly, while the influence of these products on outcome has yet to be fully elucidated, these findings suggest a plausible role in resuscitation of the equine patient, particularly when faced with a case refractory to other treatments.

### Side Effects of Colloids

Recent reports in the human literature have brought to light concerns regarding the potential for complications following synthetic colloid use. In conjunction with the now limited reliable data supporting their use, this has led to a significant move away from their use in human medicine (85). The primary side-effects seen in people that have been discussed to date include: allergic-type reactions, accumulation in the tissues; renal compromise resulting in an increased need for renal replacement therapy; and coagulation derangements resulting in a need for blood transfusion (85). Both renal and coagulation side effects may be more likely with larger molecular weight HES solutions associated with clogging glomeruli and interfering mechanically with coagulation.

In veterinary species, side-effects of colloid administration have not been well-studied. There appear to be no reports of hypersensitivity reactions and tissue accumulation. However, there has been some evaluation of their renal and coagulopathic consequences. Administration of HES did not appear to increase the risk of acute kidney injury according to one study in cats, but the two studies in dogs have divergent outcomes related to risk associated with HES administration (108–110). Changes in coagulation have been documented *in vitro* in cats and in dogs with naturally occurring hemoperitoneum (111, 112).

While there is a paucity of research regarding other sequelae following their use in horses, there is some support for changes in coagulation parameters following synthetic colloid administration (89, 104). The previously reported effects of synthetic colloids on coagulation have been attributed to both a dilutional effect and an interference with platelets, clotting factors, and the fibrinolysis system (113, 114). Based on findings reported in people, Epstein et al. evaluated the effects of two formulations of HES products – hetastarch (HS) and tetrastarch (TS) - as well as normal saline, on various hemostatic parameters in healthy horses (89). The study identified a variety of statistically significant changes in coagulation testing. It should be recognized that most changes were mild, with values often remaining in the normal range and unlikely to be clinically significant. It was found that all three solutions resulted in a transient decrease in platelet count and activated clotting time, as well as a transient increase in prothrombin and activated partial thromboplastin times (PT and aPTT, respectively). Interestingly, aPTT was higher in horses receiving HS vs. TS. Platelet aggregation was also altered in response to colloid therapy, with automated platelet function testing closure times prolonged in both colloid groups, again more so with HS than TS. Another study performed by Vilojen et al. evaluated the hemostatic effects of TS administered at varying doses (10, 20, and 40 ml/kg) in healthy horses (104). All thromboelastography (TEG) values remained within normal limits in all groups throughout the study. However, the higher dose used was considered more likely to induce measurable changes in TEG parameters. While neither of these studies were performed in clinical patients and no clinical bleeding abnormalities were reported, they do seem in line with coagulation derangements seen in other species. In contrast to these studies, no alterations to coagulation were identified following administration of hypertonic saline and HES to horses administered endotoxin under general anesthesia (115). Additional work is needed to evaluate these effects in clinical patients, particularly those more likely to be at risk for pre-existing thrombocytopenia, thrombocytopathia or coagulopathy.

## Conclusions

It is clear that, despite its frequent use, the most appropriate way in which to utilize fluid therapy is far from well-understood. Review of the literature suggests that many of the issues that plague equine clinicians mirror those encountered in human medicine. However, while multiple experimental studies have been performed, the equine literature is severely lacking in large-scale, well-designed studies in the clinical patient. This significantly limits the ability to provide practitioners with evidence-based recommendations for fluid therapy in horses. Based on the review of the present literature in horses and extrapolation from literature in other species the following basic summary points can be made:

1. IV fluid therapy remains the most appropriate route for volume resuscitation, however, IG and PR fluid administration are efficacious and seemingly safe routes for correcting and maintaining systemic hydration and increasing fecal water content at rates that have been reported in the literature.
2. Aggressive IV fluid rates (greater than twice maintenance) are likely to be no more effective at increasing fecal water than lower rates, despite significantly increasing the cost and increasing the risk of electrolyte abnormalities and volume overload.
3. Use of non-sterile isotonic fluid options may be ill-advised due to the potential for mixing errors, bacterial and endotoxin contamination, and increased risk for catheter site complications.
4. Normal saline has been repeatedly reported to result in hyperchloremic metabolic acidosis, which in other species has been associated with complications and possibly poorer outcomes. As such, balanced electrolyte solutions are likely preferable in the majority of patients. Exceptions include situations in which these effects may be therapeutic (i.e., in cases of hypochloremic metabolic alkalosis). There are more debatable benefits in cases of hyperkalemia.
5. While the proposed mechanism by which colloids exert their effects has been questioned, the equine literature to date does support their influence on colloid oncotic pressure and volume expansion.
6. Synthetic colloid administration has been shown to exert some, albeit mild, effects on hemostatic parameters similar to those seen in people and small animal patients, particularly at higher doses. As such, it may be wise to limit their administration to lower doses. It is important to note that there is no data from which to evaluate the potential for additional side effects seen in other species.

As can be seen throughout this review, with respect to evaluating fluids in the context of being a drug, the preponderance of the equine literature has focused on the first two “Ds” - drug and dosing - but very little has been evaluated with respect to duration or de-escalation. Work in other species has shifted to consideration of how these factors influence outcome, with particular emphasis on concerns such as fluid overload (68). This has resulted in a move toward consideration of fluid therapy not as a sole treatment modality with a prescribed drug, dose, and route, but rather a therapeutic intervention with 4 phases: the resuscitation phase, the optimization phase, the stabilization phase, and the evacuation phase (2, 68). Largely, to date, the average equine practitioner has likely considered the resuscitation and stabilization phases, but probably overlooked the later phases. However, some clinical research has recently been performed evaluating the possible implications of aggressive fluid therapy – such as the aforementioned study examining the effect of high IV fluid rates on intestinal hydration and another evaluating the relationship between IV fluid administration and development of post-operative reflux (36, 69). It is encouraging to see a move toward this type of thinking and greater consideration of the implications of failing to reflect on these repercussions for the patient.

## Author Contributions

The authors contributed equally to the this manuscript through researching the topic and writing and editing the manuscript. All authors contributed to the article and approved the submitted version.

## Conflict of Interest

The authors declare that the research was conducted in the absence of any commercial or financial relationships that could be construed as a potential conflict of interest.

## Footnotes

^1^Plasma-Lyte A Injection, Baxter Healthcare Corporation, Deerfield, IL, USA.

^2^Saline Solution 0.9%, VETone, Boise, ID, USA.

^3^Lactated Ringer Injection, Aspen Veterinary Resources Ltd, Liberty, MO, USA.

^4^Normosol-R, Hospira Inc, Lake Forest, IL, USA.

^5^Plasma-lyte 148 Injection, Baxter Healthcare Corporation, Deerfield, IL, USA.

^6^Normosol-M & 5% Dextrose, Hospira Inc, Lake Forest, IL, USA.

^7^Plasma-lyte 56 & 5% Destrose Injection, Baxter Healthcare Corporation, Deerfield, IL, USA.

^8^Veterinary 2.5% Dextrose & 0.45% Sodium Chloride Injection, Zoetis Inc, Kalamazoo, MI, USA.

^9^5% Dextrose Injection, Baxter Healthcare Corporation, Deerfield, IL, USA.

^10^Hypertonic Saline 7.2%, VETone, Boise, ID, USA.

^11^6% Hetastarch, Hospira Inc, Lake Forest, IL, USA.

^12^Pentaspan, Bristol-Myers Squibb, Montreal, QC, Canada.

^13^Voluven, Fresenius Kabi Norge AS, Halden, Norway.
